# Who benefits from healthcare spending in Cambodia? Evidence for a universal health coverage policy

**DOI:** 10.1093/heapol/czz011

**Published:** 2019-10-23

**Authors:** Augustine D Asante, Por Ir, Bart Jacobs, Limwattananon Supon, Marco Liverani, Andrew Hayen, Stephen Jan, Virginia Wiseman

**Affiliations:** 1 School of Public Health & Community Medicine, University of New South Wales (UNSW) Sydney, Kensington NSW, Australia; 2 National Institute of Public Health, Lot no 80, Street 289, Phnom Penh, Cambodia; 3 Khon Kaen University, Khon Kaen, Thailand; 4 Department of Global Health and Development, London School of Hygiene and Tropical Medicine, 15-17 Tavistock Place, Kings Cross, London, UK; 5 Faculty of Public Health, Mahidol University, Bangkok, Thailand; 6 School of Tropical Medicine and Global Health, Nagasaki University, Japan; 7 University of Technology Sydney (UTS), 15 Broadway, Ultimo NSW, Australia; 8 The George Institute for Global Health, Newtown, Australia; 9 University of New South Wales (UNSW Sydney), Kensington NSW, Australia; 10 Kirby Institute for Infections and Immunity, University of New South Wales (UNSW Sydney), Level 6, Wallace Wurth Building, High Street, Kensington NSW, Australia

**Keywords:** Equity, health financing, universal health coverage, benefit incidence analysis, Cambodia

## Abstract

Cambodia’s healthcare system has seen significant improvements in the last two decades. Despite this, access to quality care remains problematic, particularly for poor rural Cambodians. The government has committed to universal health coverage (UHC) and is reforming the health financing system to align with this goal. The extent to which the reforms have impacted the poor is not always clear. Using a system-wide approach, this study assesses how benefits from healthcare spending are distributed across socioeconomic groups in Cambodia. Benefit incidence analysis was employed to assess the distribution of benefits from health spending. Primary data on the use of health services and the costs associated with it were collected through a nationally representative cross-sectional survey of 5000 households. Secondary data from the 2012–14 Cambodia National Health Accounts and other official documents were used to estimate the unit costs of services. The results indicate that benefits from health spending at the primary care level in the public sector are distributed in favour of the poor, with about 32% of health centre benefits going to the poorest population quintile. Public hospital outpatient benefits are quite evenly distributed across all wealth quintiles, although the concentration index of −0.058 suggests a moderately pro-poor distribution. Benefits for public hospital inpatient care are substantially pro-poor. The private sector was significantly skewed towards the richest quintile. Relative to health need, the distribution of total benefits in the public sector is pro-poor while the private sector is relatively pro-rich. Looking across the entire health system, health financing in Cambodia appears to benefit the poor more than the rich but a significant proportion of spending remains in the private sector which is largely pro-rich. There is the need for some government regulation of the private sector if Cambodia is to achieve its UHC goals.

**Key Messages**

- Cambodia has committed to universal health coverage (UHC) and is reforming its health financing system to align with this goal. Policymakers need comprehensive evidence on who benefits from the existing health financing arrangements in order to streamline current reforms and forge a better path towards UHC.
- Benefits from health spending in the public sector are generally distributed in favour of the poor and the distribution reflects the need for health services. Private sector benefits, especially private clinics/pharmacies and private hospital outpatient departments, are substantially pro-rich.
- Cambodia has taken significant strides towards UHC but key challenges remain in ensuring that health spending delivers benefits to all Cambodians in accordance with their need for healthcare.

## Introduction

Cambodia is a lower-middle income country with a population of 15.7 million and a gross domestic product (GDP) per capita of $1269 in 2016 (World Bank, 2018b). During the last 20 years, Cambodia has witnessed significant economic development with an average annual growth rate of about 7.7% which has seen the country transition from low- to middle-income status in 2016 (World Bank, 2018a). Although most Cambodians are still economically vulnerable, with 70% of the population living on less than $5.5 a day, the proportion of people living below the national poverty line has drastically reduced from 47.8% in 2007 to 13.5% in 2014 (World Bank, 2017). Gross domestic savings as a proportion of GDP has increased from 6.6% in 2000 to 18% in 2015 (ADB, 2018; World Bank, 2018c).

Alongside this sustained economic growth, the health system of Cambodia has also seen significant improvements in the last two decades. Health outcomes have improved substantially, with life expectancy at birth rising from 58 years in 2000 to 68 in 2014, although healthy life expectancy remains relatively low at 58.9 years (UNDP, 2016). Other health indicators, including maternal and infant mortality, have markedly improved. For example, maternal mortality has declined from 472 per 100 000 live births in 2005 to 161 per 100 000 in 2015 (National Institute of Statistics *et al.*, 2015; UNDP, 2016). Childhood immunization coverage has expanded with 81% of children aged 12–23 months immunized against measles in 2016 compared with 52% in 2002. This has contributed to a steep decline in infant and under-5 mortality rates, dropping from 45 and 54 per 1000 live births in 2010 to 27 and 35 per 1000, respectively, in 2014 (National Institute of Statistics *et al.*, 2015).

Despite these achievements, health outcomes in Cambodia still rank among the poorest in the Southeast Asian region. For example, the 2015 maternal mortality rate (161 per 100 000 live births), compares poorly with rates in neighbouring Thailand (20 per 100 000 live births) and Vietnam (55 per 100 000 live births; UNDP, 2016). Malnutrition remains a challenge with about 32% of children under 5 (about 500 000 children) stunted and 9% severely stunted (National Institute of Statistics *et al.*, 2015). The country’s rate of immunization against measles—around 81% of children aged 12–23 months in 2016—lags behind those of Myanmar (91%), Vietnam (99%) and Thailand (99%; World Bank, 2018b). Additionally, like many low- and middle-income countries (LMICs), Cambodia faces a double burden of infectious and non-communicable diseases. While malaria, tuberculosis and HIV infections are still widespread, the growing burden of diabetes, hypertension and hypercholesterolaemia in the adult population is putting strain on the health system (Chhoun *et al.*, 2017).

Underlying these unfavourable indicators is the problem of limited and unequal access to quality healthcare and a dominant unregulated private health sector. Many Cambodians, particularly the poor and those living in rural areas, do not have access to quality health services despite past efforts and progress made in expanding access (Liverani *et al.*, 2017). For example, while nearly 96% of deliveries in the urban Phnom Penh province occur in health facilities (public and private), only about 46% of deliveries in the rural Kratie province occur in health facilities. Around 53% of deliveries in the Kratie province occur at home and are attended largely by unskilled birth attendants (National Institute of Statistics *et al.*, 2015).

Cambodia, like many LMICs, has committed to universal health coverage (UHC) and is implementing reforms to expand access to quality services while maintaining the gains made across the health system over the past years (Wiseman *et al.*, 2017). UHC requires nations to ensure that all citizens have access to the health services they need without the risk of financial hardship (Kutzin, 2013). An essential component of UHC is removing financial barriers to accessing health services and ensuring that people are not impoverished as a result of using healthcare. UHC-focused reform typically incorporates strategies to boost overall funding for the health system, increase the proportion of resources channelled through pooled funding such as publicly funded insurance schemes, diverting spending to services known to be effective, and ensuring equitable financial access (Kutzin, 2013; Ensor *et al.*, 2017).

Over the last decades Cambodia has implemented key health financing reforms designed to promote equity in access to effective and affordable healthcare, especially for the poor. Central among these is the country’s Health Equity Funds (HEFs), a third-party payer mechanism designed to remove financial barriers to accessing public health facilities through reimbursement of fees to facilities for health services rendered to the poor (Jacobs *et al.*, 2007; Ensor *et al.*, 2017; Wiseman *et al.*, 2017). This payer mechanism (the HEF) applies only to the public sector, which comprises 40% of health spending (government plus donor). Currently, there are no social health insurance mechanisms for the private sector. The HEF currently covers around 3.2 million Cambodians (about 20% of the population; Ministry of Health, 2016a). Other financing reforms and programmes include internal contracting of health services (Vong *et al.*, 2018), a government midwifery incentive scheme designed to boost facility deliveries (Ir *et al.*, 2015) together with the now redundant voluntary health insurance schemes targeting the informal sector (Annear *et al.*, 2011) and a range of voucher schemes intended to increase the uptake of reproductive and safe motherhood services by poor rural communities (Ensor *et al.*, 2017; Wiseman *et al.*, 2017).

Evaluations of the HEF have produced mixed results with some studies suggesting improvements in access to health services by the poor while others showing no such effect. For e.g. Noirhomme *et al.,* (2007) found that HEF enhanced access to hospital services for the poor. Dingle and Powell-Jackson reported that exposure to HEF was associated with a 4% increase in the probability of receiving free care at any health provider but no association between HEF and utilization of health services was found (Dingle and Powell-Jackson, 2016). Flores and associates found that while HEF reduces the probability of resorting to the private sector, it has no significant effect on the use of public care (Flores *et al.* 2013). The 2014 Demographic and Health Survey found that only 3.9% of men and 4.0% of women had their health services paid for by a HEF (National Institute of Statistics *et al.*, 2015). While these insights are useful, they do not offer conclusive evidence on the impact of the HEF and very little is known about the effects of other interventions beyond the HEF as no comprehensive evaluation of the equity of the health financing system in totality has been undertaken. Policymakers need comprehensive evidence on who benefits from the existing health financing arrangements in order to streamline current reforms and forge a better path towards UHC. This article assesses how the benefits from health spending are shared across different socioeconomic groups in Cambodia.

**Box 1. Overview of the health financing system**

Cambodia spends around 6% of its GDP on health, which is slightly higher than the 4.6% average spent by countries in the Southeast Asian region in 2015 (World Bank, 2018b; WHO, 2018). Total health expenditure (THE) was estimated at about US$1 billion in 2014, translating to around US$68 per capita. There are three main sources of financing for the health system—out-of-pocket (OOP), government and donor payments. OOP spending is by far the largest source of funding for the health system, constituting around 60% of THE. In per capita terms, every Cambodian contributed around US$43 in OOP health spending in 2014. However, high OOP payments are not unique to Cambodia. Many countries in the region derive more than 50% of their the from direct OOP spending (OECD/WHO, 2016). Government funding constitutes about 20% of THE and compares poorly with the 36.1% average spent by governments across lower-middle income countries (World Bank, 2018b). Donor funding accounts for the remaining 20%.

In terms of composition, Cambodia’s OOP spending is largely made up of spending for private sector services at pharmacies and clinics. In the public sector, official user fees raise funds principally to support operational costs at government hospitals and health centres, though they comprise only a small proportion of government revenue. User fees were introduced as part of health sector reforms initiated in 1996 with the objective of raising additional revenue to improve quality of services and increase staff motivation (Jacobs *et al.*, 2018; Ministry of Health, 2015a). To lessen the negative effects of user fees for government health services, Cambodia introduced the HEF to offer some degree of financial risk protection to the poor and to stimulate the use of public health services. Beneficiaries of the HEF are identified either through the national Identification of Poor Households Program (IDPoor) carried out by the Ministry of Planning or through post-identification, which is used at referral hospitals to identify poor patients who have not been pre-identified (Annear *et al.*, 2016). The HEF has expanded over time and reviews suggest, on average, beneficiary households have reduced their OOP spending on health care and seek care less frequently in the private sector (Flores *et al.*, 2013). Despite this, the overreliance on direct payment to finance healthcare in Cambodia still poses a significant challenge to the country’s desire to move towards UHC as there is some evidence that a substantial proportion of HEF beneficiaries still initiate healthcare seeking at private health providers where they incur considerable OOP expenses (Jacobs *et al.*, 2018).

Government health expenditure in Cambodia constituted about 6.1% of general government expenditure in 2015, which was below the 8.5% regional average for Southeast Asia (WHO, 2018; World Bank, 2018b). The government allocates its funding for health largely as a regular annual budget for health activities via Ministry of Health (MOH) and other health institutions. However, it also allocates funds to co-finance the country’s pooled funding arrangements with selected donors under the Health Equity and Quality Improvement Program (H-EQIP; Ministry of Health, 2018). In terms of levels of expenditure, about 30% of the 2014 government health budget was allocated to provincial levels while the rest was managed at the central MOH level (Ministry of Health, 2015a).

The health system of Cambodia relies heavily on donor (external) funding. In 2015, the average external health expenditure per capita for lower-middle income countries was US$2.6 but in Cambodia it was US$13.3—about 512% higher, and a rise of more than 831% from US$1.6 in 2000 (World Bank, 2018b). Donor health spending has also seen a small reduction. Data from 5-year Cambodia National Health Account 2012–16 indicate that general government health expenditure as a proportion of THE averaged 21% over the 5-year period; donor funding for the same period averaged 18%, while OOP spending averaged 61%. Traditionally, donor funding for health has been allocated largely as earmarked funds for disease-specific national programmes such as malaria control or the HIV/AIDS programme. However, under the H-EQIP, donors in Cambodia contribute 40% of finances required to fund the HEF while the government fund the remaining 60% (Annear *et al.*, 2016). With the continuing growth of Cambodia’s economy and maturation to middle-income status, it is expected that many donors will reduce or withdraw their health sector funding support. Global health initiatives, such as the Global Fund or GAVI, the vaccine alliance, have already requested increased government co-financing (Cantelmo *et al.*, 2018).

## Materials and methods

We employed benefit incidence analysis (BIA)—one of the standard measures of equity in health financing—to assess the distribution of healthcare benefits across different wealth quintiles. BIA is an analytical technique for measuring the extent to which different socioeconomic groups benefit from public spending for health through their use of health services (O’Donnell *et al.*, 2008; McIntyre and Ataguba, 2011; Wagstaff, 2012). A combination of primary and secondary data was used in this analysis.

The primary data were gathered through a nationally representative cross-sectional household survey involving 5000 randomly selected households across Cambodia and nearly 25 000 individuals. The survey was conducted between November 2015 and February 2016 and consisted of a sample of 1000 urban and 4000 rural households. Full details of the sampling procedure are published elsewhere (see Wiseman *et al.*, 2017; http://gh.bmj.com/content/2/1/e000153). The household survey gathered information on utilization of various types of health services including selected preventive healthcare services (family planning services, antenatal care, vaccination services, etc.), the costs incurred for using these services, and household living standard data to enable the ranking of households by their socioeconomic status. The survey also included questions on self-assessed health status for the purpose of assessing the health needs of households’ members. We used a standard recall period of 1 month for utilization of outpatient care and 12 months for inpatient and preventive care (WHO, 2011).

The secondary data were extracted from three main sources: the National Health Accounts 2014, Demographic and Health Survey 2014 and Annual Health Statistics Report 2012 (National Institute of Statistics *et al.*, 2015; Ministry of Health and WHO, 2012; Ministry of Health, 2015b). We extracted data on THE for various types of facilities (health centres, public hospital outpatient, private pharmacies, private hospital/clinics and private hospital inpatient care) from the National Health Accounts. This was matched with health service utilization data estimated from the Annual Health Statistics Report and the Demographic and Health Survey to compute the unit costs of outpatient and inpatient services in public and private facilities. We used data from the Demographic Health Survey to estimate utilization of private outpatient care (pharmacies and clinics) as the Annual Health Statistics of the Ministry of Health had no data on the use of private facilities.

The estimation of unit cost was based on the constant unit cost assumption, where each type of care (e.g. each hospital outpatient visit) is assumed to cost the same, and equal to total costs incurred in providing the type of service (i.e. subsidies plus user fees) divided by the number of units of utilization (World Bank, 2012a). To estimate the unit cost for each sub-type of service, we divided the total expenditure for that type by total utilization. We obtained disaggregated data for both public and private hospital outpatient and inpatient care from the relevant official who was involved in the compilation of the 2014 National Health Accounts report. The same unit costs for public hospital outpatient and inpatient care were applied to all hospitals regardless of the type of hospital. We adjusted the utilization data for public hospital outpatient and inpatient care obtained from the 2012 Annual Health Statistics Report to bring them to 2014 levels to match the 2014 expenditure data from the National Health Accounts.

The private hospital inpatient care was divided into private-for-profit and private-not-for-profit facilities. There was information on total expenditure for private-for-profit inpatient care but no utilization data could be found. We therefore estimated utilization of private inpatient care based on hospitalization in public facilities. In 2012, there was a total of 653 434 inpatient discharges and 6579 deaths, giving a total of 45 discharges per 1000 persons and a mortality rate of 4 per 10 000 persons. This information was used to estimate total admissions per year for public inpatient departments and a ratio of one admission in the public sector to 0.5 inpatient admission in the private sector was applied (Ministry of Health and WHO, 2012). We triangulated the estimated hospitalization data with the actual private-for-profit hospital utilization information from our household survey. The estimated use was higher than the actual data by a factor of 1:1.57 but overall rate of utilization (both actual and estimated) was low compared with private outpatient use.

In the analysis, we sought to ascertain whether the distribution of benefits from healthcare spending for a given facility was pro-poor or pro-rich and was in line with the need for healthcare. We constructed an asset index to proxy socioeconomic status using the household living standard variables in principal component analysis (Vyas and Kumaranayake, 2006; O’Donnell *et al.*, 2008). Data for outpatient visits were annualized to obtain visits per year. Self-assessed health status by households was used as a proxy of health need (Manderbacka, 1998). Respondents in the survey were asked to rate the health status of each member of their households on three response categories: ‘good’ (rarely gets ill), ‘fair’ (occasionally gets ill) and ‘bad’ (chronically and/or frequently ill). We followed the approach used by earlier studies in LMICs (Ataguba and McIntyre, 2012b; Chuma *et al.*, 2012) and classified individuals into two groups of need: good health (suggesting no need for care) if the health status of a household member is assessed as ‘good’ and poor health (suggesting need for care) if health status is assessed to be ‘fair’ or ‘bad’. To determine the amount of benefit we multiplied the unit cost per service for a given facility by the rate of usage and deducted any OOP cost incurred by the user (Asante *et al.*, 2014). A concentration index (CI) was generated and used to measure the pro-poorness of the distribution of benefit. The CI ranges from −1.0 to +1.0 and captures the degree to which health payments are distributed among the economically worse off as compared with the better off (O’Donnell *et al.*, 2008). We also presented the results using concentration curves.

## Results

### Healthcare needs and treatment seeking behaviours

Close to 36% of the study population reported being injured or sick in the last month before the survey (Table 1). Those who have visited a health facility in the past month (*n* = 8587 or 96.5% of those who reported injured or sick) were slightly lower than those who reported being injured or sick ahead of the survey. Some of these individuals may have visited a health facility for preventive purposes and not necessarily because they were sick. The overall rate of utilization of preventive healthcare (about 10.7% of individuals) appears low but given the narrow definition of preventive care in this study, covering only preventive maternal and child healthcare, this may not be as low as it seems. Besides, the use of preventive services such as family planning is very personal, and hence, the respondent of the household survey may not have a full knowledge of users of such services in the household. A small number of people (*n* = 221 or 0.9% of individuals) reported not seeking care when they were unwell and then the sickness got worse. Most of these people either thought their sickness was not serious enough to seek treatment or could not afford the costs associated with using health services.

**Table 1.** Selected descriptive statistics from household survey

Data label	Number	(%)
Number of households surveyed		
Rural Cambodia	3934	(78.6)
Urban Cambodia	1073	(21.4)
Total number of persons		
Male	11 769	(47.6)
Female^a^	12 908	(52.2)
Persons reportedly injured or sick in the last month	8913	(36.0)
Persons who visited any health facility in the last month as an outpatient	8587	(96.5)
Those who visited a health centre in the last month	1228	(14.3)
Those who visited a public hospital in the last month	538	(6.3)
Those who visited private hospital/clinic in the last month	2099	(24.5)
Those who visited private pharmacy in the last month	2569	(30.0)
Persons hospitalized in the last 12 months	1307	(5.6)
Hospitalized in a public hospital	618	(47.3)
Hospitalized in a private-for-profit hospital	122	(9.3)
Hospitalized in a private-not-for-profit hospital	562	(43.0)
Persons seeking preventive care in the last 12 months	2346	(10.7)
Persons not seeking care when sick in the last 12 months and the sickness got worse	221	(0.9)

a 73 (0.3%) undetermined. Use of outpatient care had a recall period of 1 month while hospitalization and use of preventive care had a 12-month recall period.

### Utilization of health services by socioeconomic group

**Figure 1:**
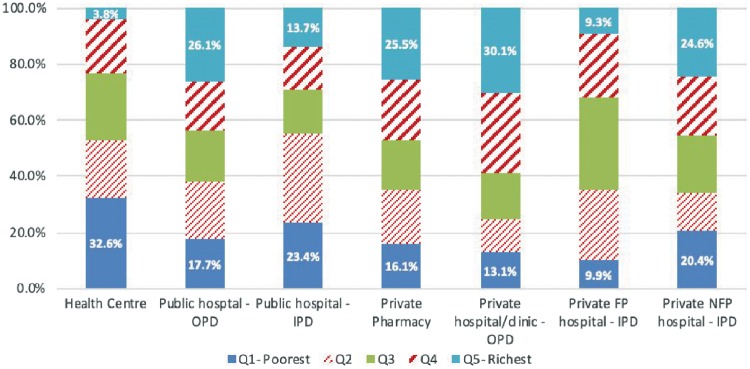
Utilization of health services by wealth quintile and facility type. *Data source*: Household survey conducted for this study. FP, for-profit; NFP, not-for-profit; OPD, outpatient department; IPD, inpatient department.

The most commonly used health services and facility sub-type in Cambodia are outpatient services in private pharmacies and private hospitals/clinics. Of those who reported visiting any health facility as an outpatient in the last month, nearly 55% visited these two facilities. Around 14.3% visited health centres (Table 1). The distribution of health service use in Cambodia, as illustrated by Figure 1, was relatively pro-poor for government health facilities and the opposite for private facilities. Health centres were mostly used by the poorest population quintile, who accounted for nearly 33% of the total health centre visits. The richest quintile accounted for just about 4% of the total health centre visits. Similarly, public hospital inpatient care was mostly used by the poor, with the bottom two quintiles using nearly 60% of all public hospital inpatient services (Figure 1). Overall, hospitalization in Cambodia, as in other LMICs, was found to be low with 5.6% of individuals reporting being hospitalized in the 12 months preceding the survey (Table 1). A little over half (53%) of all hospital admissions occurred in public hospitals. The use of outpatient departments (OPDs) in public hospitals was fairly evenly distributed across all wealth quintiles suggesting that Cambodians of all socioeconomic standing use public hospital OPDs, although the richest quintile use these services slightly more than the poorest quintile.

The distribution of the use of private health facilities was pro-rich, especially in the case of private hospital/clinics and private pharmacies services. The richest quintile accounted for about 30% of all private hospital outpatient visits compared with only 13% of the bottom quintile using these services. A similar utilization pattern was observed for private pharmacy services, where the top quintile accounted for around 26% of visits compared with 16% for the bottom quintile. Hospital admissions in the private sector were relatively evenly distributed, especially when compared with private outpatient visits—private hospital/clinics and private pharmacies. However, hospitalization in private-for-profit hospitals was neither pro-poor nor pro-rich; it appears both the richest and poorest quintile avoid the use of these facilities. Many of the hospitalizations in the private sector occurred in non-profit facilities which, in this study, accounted for 38.4% of all hospital admissions.

### Unit cost of service by facility type (in US$)

We obtained the unit cost of each type of service by dividing the total costs incurred in providing the service over a period of 1 year by the total number of units of utilization of that service (*see page 4 for sources of data used to compute unit costs*)*.* The unit costs of service for health centres was around US$2.87 and about $2.00 lower than the unit cost of service in private pharmacies (∼US$4.86; Table 2). The unit cost result for health centres is consistent with what was reported in a recent costing study in Cambodia which determined the cost per health centre visit to be US$3.24 (Flessa *et al.*, 2018). However, the public hospital OPD unit cost of US$35.16 was higher than the US$5.87 and US$9.65 Flessa and colleagues reported for a Complementary Package of Activities (CPA) 1 and CPA 2 hospitals but lower than the US$41.53 they reported for CPA 3 hospitals. The unit cost for inpatient care in the public sector was US$195.90 compared with the $162.74 in the private-for-profit sector. The difference might be due to the gaps in data for utilization of private hospitals; the Ministry of Health had no data for utilization of private hospitals, so utilization was estimated based on utilization of public hospitals. The unit cost for private-not-for-profit hospitals ($17.45) appears low. While these are mainly NGO facilities that often provide services at low cost (Alam and Ahmed, 2010), the low unit cost here may have resulted from gaps in the expenditure and utilization data used.

**Table 2.** Unit cost of health service by type of facility (US$)

Public facilities	USD
Health centre	2.87
Hospital OP	35.16
Hospital IP	195.90
Private facilities	
Private pharmacies	4.86
Private hospital/clinics OP	16.16
Private-not-for-profit hospital IP	17.45
Private-for-profit hospital IP	162.74

*Data sources:* National Health Accounts 2014; Annual Health Statistics 2012. Demographic and Health Survey 2014.

OP, outpatient; IP, inpatient.

### Distribution of healthcare benefits

The share of healthcare benefits received by different wealth quintiles as presented in Table 3 indicates a relatively pro-poor distribution in public sector facilities and a pro-rich distribution in private facilities, if private-not-for-profit facilities are excluded. Health centres and public hospital inpatient benefits were the most pro-poor with the poorest quintile accounting for 31.0% and 33.6% of benefits, respectively, compared with the 2.2% and 11.6% of these benefits going to the richest quintile of the population. The fairly high concentration index (CI) of -0.280 for health centre and -0.276 for public hospital inpatient facilities confirm the pro-poorness of the distribution. In the case of health centres, there was almost no difference between the share of health care benefits to the poor quintile (31.0%) and their share of utilization (33%). The benefit incidence for public hospital OPDs was marginally pro-poor with a CI = -0.058 although the two poorest quintiles received benefits slightly in excess of their population shares.

**Table 3.** Distribution of THE and healthcare benefits by facility type

Type of facility	Share of THE 2014 (million USD)	Percentage shares	Share of healthcare benefit (%)					CI
			Q1	Q2	Q3	Q4	Q5	
Health centre	60.2	5.8	31.0	21.9	20.1	24.9	2.2	−0.280
Public hospital OPD	303.0	29.4	21.0	23.1	19.6	17.5	18.8	−0.058
Public hospital IPD	161.0	15.6	33.6	26.2	21.7	7.0	11.6	−0.276
Total—public sector	524.3	50.9	24.6	25.0	20.4	15.9	14.1	−0.614
Private pharmacy	140.4	13.6	14.3	17.2	17.7	24.2	26.6	0.076
Private hospital/clinic OPD	343.1	33.3	10.4	11.1	16.5	33.3	28.7	0.204
Private-for-profit hospital IPD	21.5	2.1	13.6	21.8	21.6	31.4	11.5	−0.038
Total—private sector (excl. non-profit)	504.9	49.0	11.7	14.4	16.7	32.7	24.5	0.242
Private NFP hospital IPD	0.7	0.1	87.0	2.6	1.7	3.8	5.0	−0.529
Total—all sector	1.030	100.0	18.3	19.8	18.6	24.2	19.2	−0.901

*Data sources:* THE data from the National Health Accounts 2014. Data on health care benefits are derived from a household survey conducted for this study. Recall period for outpatient visits was 1 month while that of inpatient care was 12 months.

**Figure 2.**
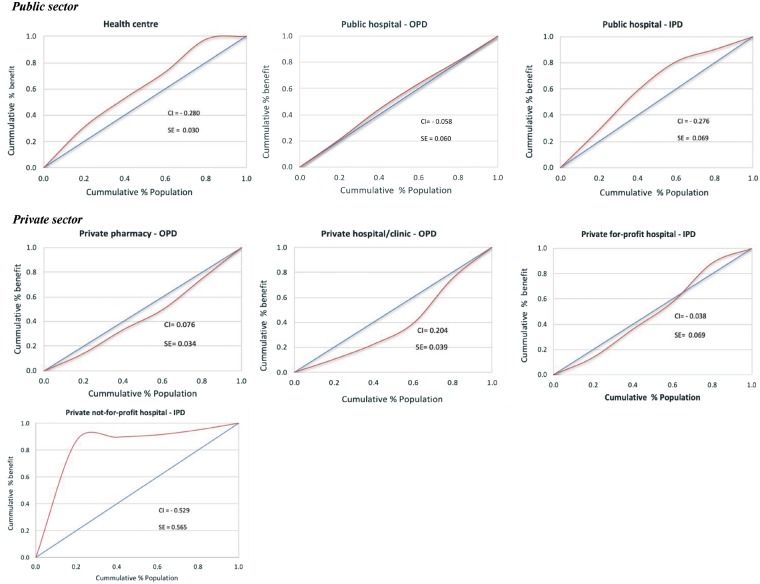
Distribution of healthcare benefits by facility and wealth quintile (concentration curve). *Data sources*: These graphs were constructed with data from the household survey conducted for this study. Unit costs data were extracted from three main secondary sources: National Health Accounts 2014; Demographic and Health Survey 2014; Annual Health Statistics Report 2012. CI, concentration index; SE, standard error; OPD, outpatient department; IPD, inpatient department.

The distribution of healthcare benefits in the private sector favoured the rich despite private hospital inpatient care being pro-poor. Outpatient care in private hospitals/clinics, which accounted for 33.3% of benefits had a strong pro-rich distribution with the richest quintile of the population capturing nearly 29% of benefits compared with only 10.4% for the poorest quintile. The concentration curve for private hospital/clinics lies considerably below the line of equality (Figure 2). A similar pattern of pro-rich distribution of benefits was observed in private pharmacies with the richest quintile accounting for 27% of benefits compared with 14.3% for the poorest quintile. Benefit incidence for private inpatient care was surprisingly pro-poor, especially in the non-profit sector where the poorest quintile received almost all the benefits—more than four times their population share (87.0%) and a high negative CI of -0.529. The concentration curve for this facility lies well above the line of equality (Figure 2). However, inpatient care in the non-profit sector in Cambodia accounted for <1% of total health spending and a small number of admissions per year. The private-for-profit sector also had a pro-poor distribution of benefits with a negative CI of -0.038. Looking across the entire health system, health financing in Cambodia appears to benefit the poor more than the rich but a significant proportion of spending remains in the private sector which is largely pro-rich. The high overall negative CI of—0.901 should be interpreted with great caution—this was driven mainly by a high negative CI for the non-profit hospital sector which accounted for <1% of total spending and by the very pro-poor public sector distribution of benefits. It does not mean the poor in Cambodia are protected against OOP payments for health.

### Distribution of healthcare benefits relative to need

**Figure 3.**
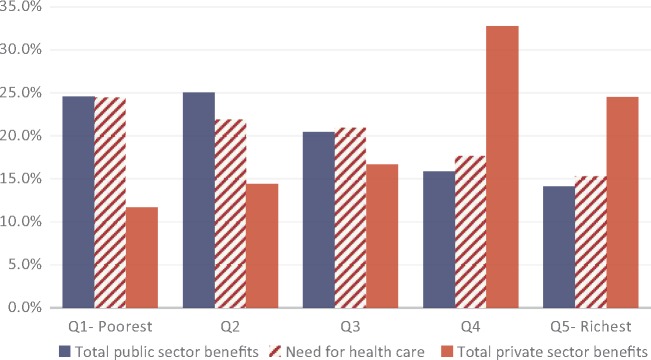
Distribution of benefits and need for healthcare.

Overall, there were only limited variations in self-assessed need across the wealth quintiles, although the level of need appears to increase with socioeconomic status (Figure 3). The poorest quintile of the population reported the highest proportion of health need, 24.4% compared with 15.3% for the richest quintile. The distribution of total healthcare benefits in the public sector was largely in line with the need for healthcare. For example, the poorest quintile with 24.4% of need received 24.6% of healthcare benefits while the richest quintile with 15.3% of need accounted for 14.1% of benefits. The distribution of benefits in the private sector was the direct opposite of the distribution in the public sector—the richest quintile with less self-assessed need captured the largest proportion of benefit—24.5%—which was significantly higher than their share of need. Of note, the two richest quintiles together accounted for nearly 60% of the total private sector benefits. In contrast, the poorest quintile received a relatively lower share of total private sector benefits (11.7%) despite having the highest level of need.

## Discussion

The results presented above show that benefits from health spending in the public sector in Cambodia are generally distributed in favour of the poor and the distribution reflects the need for health services. The distribution of benefits was mostly pro-poor at the health centre level which is consistent with findings reported elsewhere in other LMICs. For example, Asante *et al.* (2017) found a pro-poor distribution of benefits across health centres in Fiji. BIA studies undertaken in Southeast Asian countries such as Thailand, Malaysia and Vietnam have all reported a pro-poor distribution of PHC benefits (Yu *et al.*, 2008; Limwattananon *et al.*, 2012; World Bank, 2012b). While such distribution is critical for UHC, the limited funding often allocated for service delivery at the health centre level can affect service quality and undermine the overall effort to improve health outcomes under UHC. Over 80% of THE in Cambodia, as observed in this study, is expended on secondary and tertiary care, leaving just around 20% of expenditure for PHC services (public and private). Increasing funding for PHC services will not only improve coverage but also has the potential to quality of services delivered and attract more patients to use these services (Shaw *et al.*, 2015).

At the hospital level, the marginally pro-poor distribution of outpatient benefits in the public sector (CI = -0.058) is consistent with what has been reported in other settings including neighbouring Thailand (Limwattananon *et al.* 2012) and Fiji (Asante *et al.* 2017). However, in many other countries, including Kenya (Chuma *et al.* 2012) and Mongolia (World Bank 2012c), public sector outpatient benefits have been pro-rich. In their systematic review of equity in healthcare financing in LMICs, Asante *et al.*, (2016) found 12 studies from the Asia-Pacific region that reported a pro-rich distribution of benefits for hospital OPDs. The pro-poor distribution in Cambodia may have been facilitated by the country’s HEF, which has ensured some access to hospital care for the poor. Indeed, a recent study by Annear and colleagues (2016) found that >20% of services at referral hospitals and health centres are supported by the HEF, and based on this, the authors concluded that HEF members (the poor) have greater access to hospitals and health centres relative to their population size.

The distribution of inpatient care benefits in Cambodia is inconsistent with global trends. While the results of this study indicate a clear pro-poor distribution of benefits for public sector inpatient care, such benefits have been distributed in favour of the rich in many LMICs. For example, in Ecuador, Angeles and associates (2007) found the distribution of inpatient care to be pro-rich enough to neutralize the pro-poor distribution of PHC benefits and brought the overall distribution down to proportional. Pro-rich distribution of inpatient care has also been reported in Ghana (Akazili *et al.*, 2012; Mills *et al.*, 2012), India (Chakraborty *et al.*, 2013) and Indonesia (O’Donnell *et al.*, 2007). The Cambodia result gives some indication that access to hospital services for the poor may be improving.

Improved access to hospital services for the poor is vital for vertical equity and needs to be further strengthened. Jacobs *et al.* (2018) have observed that even with the improved access, the use of public sector health services by poor Cambodians who are eligible for HEF is still low, suggesting the system could be made even more pro-poor if it were able to attract more current HEF beneficiaries. However, it is important to ensure that the hospital referral system functions at the optimum level to avoid referral of cases that can be handled at the health centre level. Inappropriate referrals can encourage HEF members to bypass health centres as they become used to free treatment at the hospital level. Strengthening the PHC system, including the quality of services will also help reduce unnecessary referrals.

The distribution of total benefits for private health facilities (excluding non-profit facilities) was overall pro-rich and driven largely by a relatively strong pro-rich distribution of outpatient benefits (private pharmacies and private hospital/clinics). These findings confirm what has been reported in several countries including Kenya, Ghana and Tanzania (Chuma *et al.*, 2012; Mills *et al.*, 2012; Mtei *et al.*, 2012). In Southeast Asia, Limwattananon and colleagues (2011) also reported pro-rich distribution of outpatient benefits in private facilities in Thailand. The pro-rich distribution in the private sector per se is not a bad thing—after all, if the rich can pay to use private care, it may free up resources for a more equitable and better quality care in the public sector. The challenge for Cambodia, however, is that many poor people still pay OOP in private facilities (National Institute of Statistics *et al.*, 2015). Outpatient services in private pharmacies and private-for-profit hospitals/clinics account for nearly 47% of benefits. Given the sector is highly unregulated and delivers services that are largely perceived to be of low quality (Ministry of Health, 2018), this may subject patients to unnecessary and expensive care which will push more poor Cambodians further into poverty and undermine the objectives of UHC. The public sector may come under further pressure as it will end up caring for those who have been ‘mistreated’ by the private sector.

The benefit incidence for private hospital inpatient care was pro-poor in this study, which is inconsistent with findings reported elsewhere. In fact, the few BIA studies that have covered the private sector have rather found the distribution of benefits for inpatient care to be pro-rich (Castro-Leal *et al.*, 2000; Limwattananon *et al.*, 2011; Ataguba and McIntyre, 2012a; Mtei *et al.*, 2012; Asante *et al.*, 2017). In the case of Cambodia, benefits were marginally pro-poor for private-for-profit facilities (CI = −0.038) but substantially so for private-not-for-profit hospitals (CI = −0.529). The not-for-profit hospital distribution is consistent with what has been reported elsewhere (Chuma *et al.*, 2012). Not-for-profit providers play a critical role in the provision of healthcare for the poor. In Ghana, the Christian Health Association of Ghana (CHAG)—a major group of health services—account for an estimated 42% of total health services in the country with about 41% of its operating budget coming from the government of Ghana (Saleh, 2013). In Cambodia, there were over 180 NGOs working in the health sector as of December 2015 (Ministry of Health, 2016b). This underscores the importance of the non-profit provider sector. Given the pro-poor distribution of benefits in this sector, it is one area the government may want to explore in terms of forming productive partnerships to boost the country’s efforts of moving towards UHC.

A key policy implication of these results is the need for some government regulation of the private health sector in Cambodia. As already indicated, the distribution of private healthcare benefits in favour of the rich is not an issue. The issue rather is the large number of Cambodians who initiate care in the private sector, where almost everything is paid OOP. Nearly 55% of individuals in this study who sought outpatient care in the last month did so in private pharmacies and private hospitals/clinics. This may undermine Cambodia’s effort to move towards UHC as sufficient financial protection cannot be guaranteed under such high initiation of care in the private sector. Government regulation can help to limit the amount of fees private providers charge and reduce the potential of catastrophic healthcare payments. Government regulation can also ensure that a certain minimum level of quality care is provided by private facilities. Although this study did not attempt to measure quality of care in the private sector, it has been recognised that low-quality care can subject patients to unnecessary and expensive treatment which may in turn push the poor further into poverty (McPake and Hanson, 2016).

### Limitations

A key limitation of this analysis is our inability to account for quality of healthcare in the BIA. The quality of health service consumed by different socioeconomic groups may differ across facilities and geographic location. The poorest quintile might be using low-quality health services compared with the richest quintile (Asch *et al.*, 2006; Sharma *et al.*, 2017). Focusing on the rate of utilization and unit cost of service to calculate benefit without considering variations in quality might lead to the overestimation or underestimation of benefits across different wealth quintiles. Another limitation is the estimation of unit cost for private-not-for-profit hospitals. Unlike other types of facilities, utilization data was not available for the calculation of unit costs.

Finally, there is scepticism about the use of subjective health measures as a proxy of health need particularly where populations are diverse and use different frames of reference for evaluation (Cheung, 1999). Despite such concerns Self-Assessed Health (SAH) has been shown to be a reliable indicator of general health and well-being across a number of different settings (Idler and Benyamini, 1997; van Doorslaer and Gerdtham, 2003), and continues to be widely used as a proxy of health need.

## Conclusion

Cambodia has taken significant strides towards UHC but key challenges remain in ensuring that health spending delivers benefits to all Cambodians in accordance with the need for healthcare. The results of this study clearly demonstrate that the benefits from health spending in the public sector in Cambodia are generally distributed in favour of the poor and this distribution reflects the need for health services. However, nearly 50% of THE remains in the private sector which distributes healthcare benefits in favour of the rich. This is a huge challenge that must be tackled if the UHC dream is to become a reality. Given that public spending on health constitutes just around 20% of THE, there is an opportunity for the government to increase its share of health spending and allocate funds strategically to achieve value for money. This will include ensuring that funds are allocated to improve quality of health services at all levels of the health system.
