# Erratum: Who benefits from healthcare spending in Cambodia? Evidence for a universal health coverage policy

**DOI:** 10.1093/heapol/czaa037

**Published:** 2020-06-03

**Authors:** Augustine D Asante, Por Ir, Bart Jacobs, Limwattananon Supon, Marco Liverani, Andrew Hayen, Stephen Jan, Virginia Wiseman

*Health Policy and Planning*, Volume 34, Issue Supplement_1, October 2019, Pages i4–i13, https://doi.org/10.1093/heapol/czz011

**Published:** 23 October 2019

**Erratum**

Following the publication of the original article in October 2019, it came to authors’ attention that few errors remained. The authors have corrected these errors as below:

***Table 3***

The errors appeared in the last column – Concentration index (CI): Total – public sector CI should be -0.120 instead of -0.614, Total - private sector (excl non-profit) 0.176 instead of 0.242; and Total – all sector CI should be 0.025 and not -0.901.

Because of these changes the following sections of the paper have been amended:

***Abstract (lines 17-19)***

Looking across the entire health system, health financing in Cambodia appears to benefit the rich slightly more than the poor with a significant proportion of spending remaining in the private sector which is largely pro-rich.

***Page i10 (right column, para 1)***

Looking across the entire health system, health financing in Cambodia appears to benefit the rich slightly more than the poor with a significant proportion of spending remaining in the private sector which is largely pro-rich. The overall positive CI of 0.025 confirms the marginally pro-rich distribution. Although the substantially pro-poor distribution in the non-profit hospital IPD sector is desirable, overall the sector is quite small, accounting for <1% of total spending.

